# A simple methodological validation of the gas/particle fractionation of polycyclic aromatic hydrocarbons in ambient air

**DOI:** 10.1038/srep11679

**Published:** 2015-07-01

**Authors:** Yong-Hyun Kim, Ki-Hyun Kim

**Affiliations:** 1 Department of Civil and Environmental Engineering, Hanyang University, 222 Wangsimni-Ro, Seoul 133-791, Korea

## Abstract

The analysis of polycyclic aromatic hydrocarbons (PAH) in ambient air requires the tedious experimental steps of both sampling and pretreatment (e.g., extraction or clean-up). To replace pre-existing conventional methods, a simple, rapid, and novel technique was developed to measure gas-particle fractionation of PAH in ambient air based on ‘sorbent tube-thermal desorption-gas chromatograph-mass spectrometer (ST-TD-GC-MS)’. The separate collection and analysis of ambient PAHs were achieved independently by two serially connected STs. The basic quality assurance confirmed good linearity, precision, and high sensitivity to eliminate the need for complicated pretreatment procedures with the detection limit (16 PAHs: 13.1 ± 7.04 pg). The analysis of real ambient PAH samples showed a clear fractionation between gas (two-three ringed PAHs) and particulate phases (five-six ringed PAHs). In contrast, for intermediate (four ringed) PAHs (fluoranthene, pyrene, benz[a]anthracene, and chrysene), a highly systematic/gradual fractionation was established. It thus suggests a promising role of ST-TD-GC-MS as measurement system in acquiring a reliable database of airborne PAH.

The presence of polycyclic aromatic hydrocarbons (PAHs) in ambient air is mostly due to anthropogenic processes, particularly incomplete combustion of organic fuels like coal, oil, and gas^1^. The International Agency for Research on Cancer (IARC) has designated some PAHs (e.g., benzo[a]pyrene (BAP), and benz[a]anthracene (BAA)) as known human carcinogens^2^. In particular, BAP is listed as a “level 1 carcinogenic substance”^2^,^3^,^4^. PAHs are present in the atmosphere in both gaseous and particulate phases. Because the concentrations of PAHs in ambient air are low, e.g., at or below several ng·m^−3^ levels, the experimental procedures for their sampling and analysis are sufficiently complicated to suffer from large uncertainties due to low recovery despite large sampling volume^5^.

In order to accurately quantify trace level PAHs in ambient air, a highly sensitive analytical system must be employed, even after acquisition of a large sampling volume. Many researchers have relied on protocols such as the US Environmental Protection Agency (EPA) method to calculate quantities from ambient air samples^6^,^7^,^8^,^9^. Accordingly, atmospheric samples should be collected using high-volume air samplers (HVAS), which allow selective collection of gaseous and particulate PAHs on a quartz (or glass) filter and polyurethane foam (PUF) sampler, respectively. In such a sampling system, a flow rate of approximately 0.1 to 1 m^3^ min^−1^ is recommended with a sampling time of about 0.5 to 3 days. The PAHs collected by the filters and PUF sampler are subjected to Soxhlet extraction with appropriate solvent, and the extract is concentrated by an evaporator. Then, the extracts are repeatedly passed through a silica column for purification (over 24 hours). The re-concentrated extracts are analyzed by gas chromatography (GC) coupled with mass spectrometry (MS) or by high-performance liquid chromatography (HPLC) equipped with a fluorescence detector (FLD)^6^,^7^,^10^. In addition to this complicated pretreatment procedure, the US EPA method further requires the use of internal standards to balance low recovery stemming from complicated extraction procedures.

In this study, a highly simplified technique for the analysis of PAH in ambient air was developed and validated using sorbent tube (ST) sampling and thermal desorption (TD)-GC-MS analysis (Table 1). We were able to accurately describe the distribution of PAH in different phases with reliable QA data and to eliminate complicated sampling and pretreatment procedures. To this end, the minimum range of air sampling volume (e.g., less than several m^3^) was first investigated in order to create optimal conditions for the PAH analysis by ST/TD-GC-MS. The reliability of each process was examined further through the assessment of basic QA parameters (detectability, recovery, and breakthrough volume). This method was employed to explore the fractionation patterns between gaseous and particulate phases from a series of real ambient samples.

## Results and Discussion

### Results of PAH calibration experiments based on the ST method

In order to assess the reliability of our ST method for PAH analysis, the calibration results of 16 target PAHs were obtained by analyzing the liquid-working standard (L-WS) using a TD-GC-MS system (Table S1). All target PAHs had fairly good linearity with R^2^ values >0.99 (mean R^2^ ± SD (n = 16) = 0.9987 ± 0.0015). The analytical precision of PAH determination was also assessed in terms of relative standard error (RSE, %) using triplicate analyses of the third calibration point of the L-WS (BAP = 4.98 ng·μL^−1^; analytical volume = 1 μL). The RSE values of target PAHs were fairly low, with a mean of 0.57 ± 0.22%. In this way, accurate and reliable QA data for PAH analysis were acquired through the ST-based analysis of liquid-phase PAH standards.

### Detectability of PAHs in terms of LOD and MDL

In this study, the detection limits (DL) of target PAHs were determined based on the ST method using the L-WS in two common ways: (1) method detection limit (MDL) and (2) limit of detection (LOD) (Table S2). Although the MDL is more representative of actual or practical detection limits than the LOD, the LOD values were commonly reported as the ultimate limit of detection (as their DL) in many previous studies of PAHs. It should however be noted that the use of the LOD values can be misleading in a practical sense, as they tend to be significantly lower than the MDL values^11^,^12^. In this research, to assess the gaps between the two contrasting DL concepts, the MDL values were also assessed according to the relevant US EPA guidelines. To this end, seven repetitive analyses were completed using a diluted L-WS (BAP = 49.8 pg·μL^−1^: 25-fold dilution of the lowest concentration standard) in order to determine the standard deviation (SD) in peak areas. The resulting SD values were then multiplied by 3.14 (Student’s t-value at the 99.9% confidence interval) and divided by the response factor (RF) to yield the MDL in mass quantity (pg). On the other hand, LOD values were determined as three times the standard deviation of background noise (n = 7). The MDL values for 16 target PAHs were found to range from 5.19 pg (BKF) to 27.0 pg (ACL) (mean 13.1 ± 7.04 pg), while the LOD values were in the range of 0.57 pg (DBA) to 1.23 pg (FLR) (mean = 0.76 ± 0.20 pg). A wide range of DL values of PAHs have been reported, from several pg to ng levels. As shown in Table 2, the DL values of this study are considerably lower than those in many previous studies.

**Figure 1:**
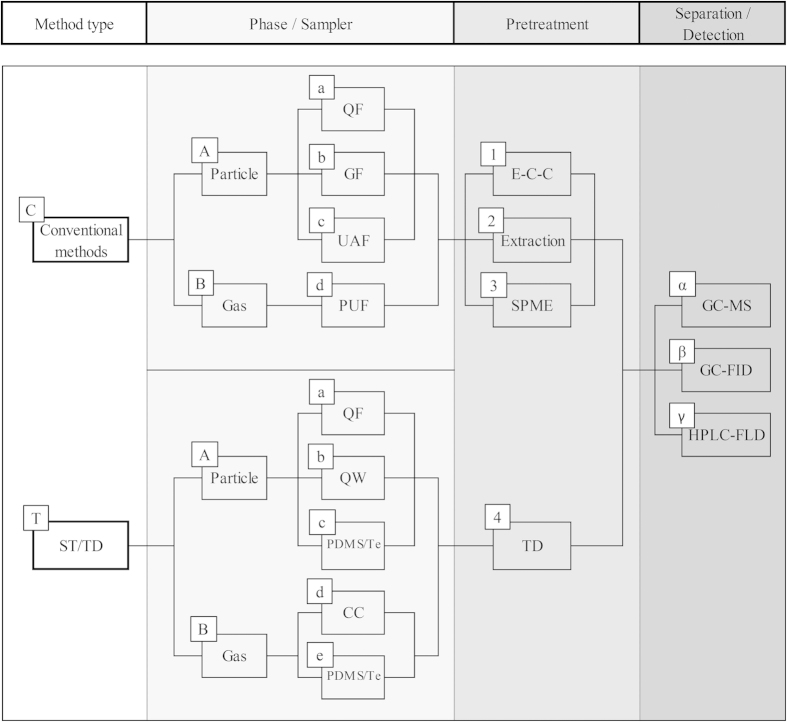
Flow chart of analytical options for the quantitation of ambient PAH between gas and particles. Abbreviation:A. Sampler (QF = quartz filter, GF = glass filter, UAF = ungreased aluminium foil, PUF = polyurethane foam, QW = quartz wool tube, PDMS = polydimethylsiloxane, CC = Carbopack C tube, and Te = Tenax TA); **B**. Pretreatment (E-C-C = Extraction-Cleanup-Concentration, SPME = Utilized direct immersion-Cold fiber-Solid phase microextraction, and TD = thermal desorption); and **C**. Separation/Detection (GC = gas chromatography; HPLC = high-performance liquid chromatography, MS = mass spectrometry, FLD = fluorescence, and FID = flame ionization detector).

For example, Ma *et al.*^13^ analyzed PAHs using the US EPA method (HVAS sampling, extraction-cleanup-concentration (E-C-C) pretreatments, and GC-MS detection) and reported MDL values of 9.21–25.3 pg, which are similar to those of the present study (the range of MDL for 16 PAHs: 5.19–27.0 pg). In contrast, using the same analytical method as Ma *et al.*^13^, others have reported MDL values one to two orders of magnitude higher (66.2–497 pg and 200–1,000 pg, respectively)^14^,^15^. Interestingly, some researchers who employed an HPLC-FLD system after the E-C-C pretreatment procedures presented LOD values of 0.58–7.99 pg and 0.70–4.30 pg, respectively^16^,^17^. As such, these HPLC-based LOD values appear to be highly comparable to our results (the range of LOD for 16 PAHs: 0.57–1.23 pg). All types of methodological options involved in sampling/pretreatment/detection are summarized in Fig. 1. In Table 3, the different detection limits are also compared using the clarification criteria introduced in Fig. 1.

### Comparison of detectabilities associated with sampling and pretreatment procedures

It is interesting to consider explanations for the observed differences in detectability of PAHs between different studies. The DL values for PAHs in this study were similar to or somewhat lower than those in many previous studies, probably due to differences in total sampling volume vs. the amount actually delivered into the detection system. Note that most previous studies generally relied on large sampling volumes (e.g., exceeding 1,000 m^3^) to quantify ambient PAHs at sub ng·m^−3^ levels (Table 3). If PAH samples are subject to the E-C-C pretreatment procedures (US EPA method), the actual mass for quantification is substantially reduced as follows. First, only certain fractions of the filter used for PAH collection are included due in the extraction. Hence this approach is subject to dual loss effects (only a small fraction of the sample is used for the extraction and subsequent loss due to treatment) that lead to a noticeable reduction in MDL under the ideal recovery conditions for PAHs at 0.01% to 2% (Table 2).

To obtain sufficient quantities of analytes (PAHs), a high-volume air sampler has commonly been employed to collect air samples of 100 to 1,000 m^3^ levels at high flow rates (>100 m^3^ min^−1^) over one to two days. In the present research, the volume size of PAH samples was reduced dramatically to 1.44 m^3^ (flow rate of 2 L min^−1^) by employing a low flow rate mini-vacuum pump. Despite this small sample volume, the absolute mass collected for each sample amounted to 17.8 pg (BKF) ~ 161 ng (NAP), which is still about 3 to 1,500 times larger than the MDL values. We are currently extending our efforts to improve ST sampling more efficiently in order to considerably shorten the sampling time for rapid monitoring of PAHs.

Bates *et al.*^18^ analyzed PAHs using a procedure similar to our TD-GC-MS method. Although they used a similar system for PAH analysis, their methods were limited in that the media used for collecting air samples (filter) and for analyzing standards (sorbent tube) were different from each other. They relied on LVAS to collect 24 m^3^ of air on a quartz filter. However, as they were unable to establish the optimal conditions of ST-TD-GC-MS (e.g., sufficiently high temperature for sample transfer in a TD system), their DL values for the PAH analysis are considerably higher than ours (mean LOD: (1) Bates *et al.*^18^ = 122 ± 69.0 pg vs. (2) This study = 0.76 ± 0.20 pg). The use of ST-TD-GC-MS system for the analysis of airborne PAHs can also be found from some other previous studies^19^,^20^. In those studies, the ST packed with Polydimethylsiloxane foam filter and Tenax TA was used to collect the PAH samples in air. In the case of Wauters *et al.*^19^, the DL values for PAHs were significantly low with mean 1.86 ± 0.79 pg (LOD, n = 16), but they did not present the MDL values. In addition, their quantitation was not made separately for each of particle and gaseous phase, as a single tube (packed with PDMS and Tenax TA adsorbent) was used to collect PAH in both phases for the TD-based analysis.

### Test of breakthrough volume of PAHs on the ST

In this study, ambient PAHs were collected on the ST using a small vacuum pump. A total of 1.44 m^3^ of air was drawn for 12 hours at an air flow rate of 2 L min^−1^. The required sample volume for PAH analysis is quite small (1.44 m^3^) compared to those in most previous studies, but the breakthrough volume (BTV) of PAHs on the ST sampler needs to be assessed for accurate quantification. To examine the BTV of PAHs on the ST, N_2_ gas was purged to the ST with six different volumes after loading the L-WS.

Table S3 shows the mass recovery of PAHs in the CC tube with different purge volumes in order to test the BTV. The PAH mass recovery was calculated using the RF values (ng^−1^) obtained by L-WS analysis: (1) Measured mass (ng) = Peak area / RF value (ng^−1^) and (2) Relative recovery (%) = Measured mass (ng) / Injected mass (ng) *100. For the total purge volume of 1 L, the recovery of PAH averaged 72.6 (±3.89%: SD). Likewise, at the low purge volume of 1 L, adsorption-partitioning equilibria were not attained between analytes and sorbents in the CC tube, resulting in poor recovery. However, if the purge volume increased above 9 L, all target PAHs had sufficiently high recoveries (>99%; mean recovery = 99.5 ± 0.50%). These high PAH recoveries were maintained up to the maximum tested purge volume of 2.52 m^3^ (mean recovery = 99.1 ± 1.29%). The results of our BT point test did not directly identify the BT but confirmed the importance of a purge step to ensure optimal recovery. As a result, we were able to predict that the BT of PAHs on the ST should not occur during routine sampling (e.g., up to 1.44 m^3^ of sample volume).

### The partitioning behavior of PAHs in air between gas and particulate phases

As a means to demonstrate the feasibility of our ST method for PAH analysis, ambient PAH samples were continuously measured using the ST method. The PAH sampling was conducted on the seventh floor of the Jae Sung Engineering Building, HanYang University, for five successive days in Oct. 2014. The QC sampler (QW + CC tubes) was used as sampling media to separately collect the particulate and gaseous PAHs from outdoor air. In addition, triplicate samples of ambient PAHs were also simultaneously collected and analyzed using three QC samplers in order to test the reproducibility (or compatibility) of the QC sampling method.

**Figure 2:**
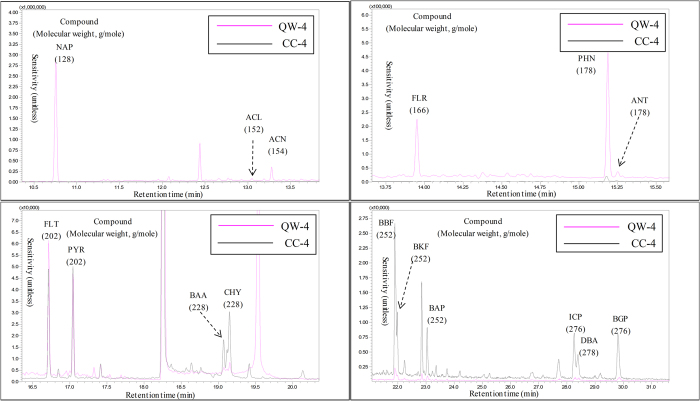
Chromatograms of the 16 PAH measured from ambient air samples collected on the fourth of five consecutive daily runs (Particle (QW-4) vs. gas phases (CC-4)).

Table 4 shows the results of the quantitative analysis of 16 target PAHs in ambient air samples collected at daily intervals. During the five sampling days, total PAH concentration (sum of the 16 PAHs) averaged 78.8 ± 38.2 ng·m^−3^ (range (n = 5): 30.1 (5th day) to 132 ng·m^−3^ (4th day)). The total PAH concentration data exhibited roughly four-fold variation during these five days. When the relative proportions of individual PAHs were compared against their total concentration, NAP demonstrated the highest value, with a mean of 66.8% ± 7.82% (Figure S1). Thus, the total PAH concentration was most strongly influenced by the NAP concentration. In contrast, five- to six-ringed PAHs had very low concentrations, typically below 1 ng·m^−3^. The results of triplicate analyses of ambient PAHs confirmed that NAP was predominant (the relative proportion of NAP = 59.1 ± 3.34%) (Table 4): total PAH concentrations of triplicate analyses (A, B, and C) were 110, 88.4, and 77.6 ng·m^−3^, respectively (mean: 91.9 ± 16.2 ng·m^−3^). Although ambient PAHs were sampled at the same time, the total PAH values varied moderately between different samples (RSE = 10.2%). However, if the NAP from these triplicate analyses was excluded from the total concentration, the compatibility between triplicates increased greatly to 40.6 (A), 37.9 (B), and 33.2 (C) ng·m^−3^ (RSE = 5.76%) (Table S4). The chromatograms of 16 target PAHs detected from outdoor air are presented in Fig. 2 using the results from the fourth day (10 Oct. 2014).

The target PAHs showed a clear partitioning pattern between gas and particulate phases, especially based on such simple criteria as the number of aromatic rings and/or molecular weight (Figure S2). The two- and three-ringed PAHs existed mainly in the gas-phase (mean gas fraction = 96.6% ± 4.01%). In the case of four-ringed PAHs, systematic fractionation was established across the particle/gas boundary. For instance, in the particle fraction, FLT and PYR (molecular weight = 202 g/mole) remained at 40.3% ± 6.90%, while BAA and CHY (molecular weight = 202 g/mole) were much more abundant (mean 77.4% ± 5.34%). All five- and six-ringed PAHs were detected predominantly in the particulate phase (mean particle fraction = 87.4 ± 4.71%). As such, the particle/gas partitioning ratio increased consistently and systematically with increasing molecular weight.

The results of particle-gas partitioning patterns in this study are thus very similar to those reported from many previous studies based on conventional methods (e.g., US EPA methods) (Table 5). Simcik *et al.*^21^ quantified gaseous and particulate PAHs in outdoor air (sampling sites: (1) Chicago (southwest and north winds) and (2) Lake Michigan (southwest and north winds, USA)) using a glass fiber filter and PUF sampler, respectively. Accordingly, the fraction of three-ringed PAHs (FLR, PHN, and ANT) in the particulate phase averaged 3.03% ± 3.01%, while those of five- and six-ringed PAHs (BBF, BKF, BAP, BGP, and benzo[e]pyrene) comprised a high proportion (mean 88.9% ± 8.90%). In the case of four-ringed PAHs, the particulate fractions of 202 g/mole (FLT and PYR) and 228 g/mole (BAA and CHY) were measured as 15.1% ± 10.4% and 60.1% ± 12.1%., respectively. Despite differences in experimental approaches compared to our study, the results of Simcik *et al.*^21^ also showed a systematic fractionation of PAHs in air to be strongly associated with molecular weight. This type of particle-gas partitioning pattern of ambient PAHs was in fact observed not only in Simcik *et al.*^21^, but also in many other previous studies. Ma *et al.*^13^,^22^ analyzed ambient PAHs at Harbin and Beijing, China using an analytical method comparable to that of Simcik *et al.*^21^ and reported that five- to six-ringed PAHs had high fractionation in the particulate phase (>99%). In contrast, the particle fractions of two- and three-ringed PAHs were relatively low, with means of 17.4% and 5.93%, respectively. Albinet *et al.*^17^ were unable to detect heavy PAHs with more than five aromatic rings in the gas fraction.

## Conclusions

In order to analyze ambient PAHs at sub ng·m^−3^ levels, large sampling volumes (and long sampling times of up to a few days) and complicated pretreatment procedures (such as extraction, clean-up, and concentration) are required. In addition, the use of an internal standard is required in order to balance low recovery stemming from the loss of analytes due to the complicated pretreatment. In this study, a novel technique for PAH analysis was developed using ST sampling coupled with a TD-GC-MS system and was validated against real ambient air samples. To this end, basic calibration and QA data for PAH analysis were acquired by ST-based analysis. Then, ambient PAH samples were collected continuously over a five day period in October 2014 using the ST and analyzed using a TD-GC-MS system. In addition, to remove the carry-over effect of the ST-based analysis, conditioning of ST was carried out in all stages of PAH analysis.

All 16 target PAHs had fairly good linearity (R^2^ > 0.99) and reproducibility (RSE < 1%) according to the ST-based analysis of the liquid-phase PAH standards. In addition, the MDL values of all PAHs as determined by the ST method were very low, with a mean of 13.1 pg. In addition to the simplicity of the ST method (without pretreatment procedures or an internal standard), it is possible to accurately quantify ambient PAHs (at sub ng·m^−3^ levels) at sufficiently low sampling volume (1 m^3^ level). For a daily ambient sample of 1.44 m^3^, the total concentration of target PAHs averaged 78.8 ng·m^−3^ over a five day period. Light PAHs were detected predominantly in the gas phase (sampled by the Carbopack C tube), while heavy PAHs existed mainly in the particulate phase (collected by a quartz wool tube). In the case of BAP, the mean of 0.21 ng·m^−3^ was detected from the ambient samples, representing a particle fractionation of 86.5%. The four-ringed PAHs showed dynamic fractionation between gas and particulate phases. This study thus successfully demonstrated the feasibility of ST-based sampling and TD-based analysis for accurate and reliable quantification of PAHs. In addition, for practical application of the ST method, we confirmed that a reasonably small (1 m^3^) sample volume is sufficient. Due to the thermal desorption procedure employed for PAH analysis in this study, we were able to simplify the pretreatment procedures for the optimum recovery of the PAHs. This ST method can thus be used to establish a routine monitoring system for PAH and to replace the methods or procedures based on conventional systems.

## Methods

### Preparation of liquid PAH standards

A total of 16 PAHs promulgated as priority pollutants by the US EPA were selected as the target analytes in this research (Table S5): (1) naphthalene (NAP), acenaphthylene (ACL), acenaphthene (ACN), fluorene (FLR), phenanthrene (PHN), anthracene (ANT), fluoranthene (FLT), pyrene (PYR), benz[a]anthracene (BAA), chrysene (CHY), benzo[b]fluoranthene (BBF), benzo[k]fluoranthene (BKF), benzo[a]pyrene (BAP), indeno[1,2,3-c,d]pyrene (ICP), dibenz[a,h]anthracene (DBA), and benzo[g,h,i]perylene (BGP). The liquid working PAH standards (L-WS) used for calibration and quality assurance (QA) were prepared by the dilution of EPA 610 Polynuclear Aromatic Hydrocarbon Mixture (Supelco, St. Louis, MO, USA) with methanol. Liquid working standards were produced to cover a relatively wide range of concentration levels (e.g., BAP = 1.25 to 49.8 ng·μL^−1^) in 2 mL vials (Table S6).

### Preparation of sorbent tubes

The ST for the collection and analysis of 16 target PAHs was prepared to calibrate L-WS and to quantify real ambient samples. The feasibility of the ST method for PAH analysis has been explored in many previous studies 18,23. However, the use of ST in those studies was confined to calibration only. Earlier researchers encountered some technical limitations due to the difficulty of increasing sampling volume with an ST sampler, although it is crucial to acquire a sufficient quantity of analytes for detection. Hence, the use of glass or quartz filters adaptable to large volume sampling (>tens of m^3^) was used in order to expand the sampling capacity of ambient air to the maximum level. Consequently, the application of ST in the TD-based analysis was confined to standard calibration experiments rather than for actual sampling^18^. In light of the physical differences in media used for environmental sampling (filter) and standard calibration (ST), the objectivity of the QA data in previous studies is somewhat questionable^18^,^24^,^25^. In the present study, to overcome diverse technical or practical problems encountered in many previous TD-based analyses, we designed a new approach to maintain the compatibility of the sampling methods by employing the same ST for both standard calibration and sample analysis.

In this study, for the collection and analysis of all target PAHs in gas and particle fractions, we prepared two types of ST. The first tube was filled with 50 mg of Carbopack C (60/80, Supelco, USA) applied to 10 mg of quartz wool (Supelco, USA) (the “CC tube”). Carbopack C was selected as the sorbent in order to induce optimal adsorption of gas-phase PAHs^26^,^27^. The second tube was packed solely with 25 mg of quartz wool (QW tube) and was used to capture particulate PAHs.

**Figure 3:**
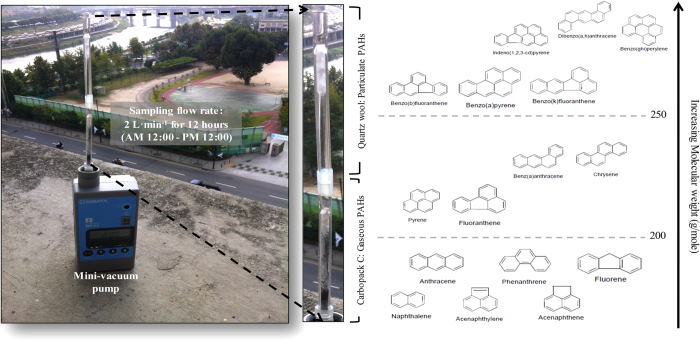
Sampling of PAHs in ambient air using a sorbent tube equipped with a vacuum pump (Sampling point: Seventh floor of the Jae Sung Engineering Building, Seoul, Korea).

**Figure 4:**
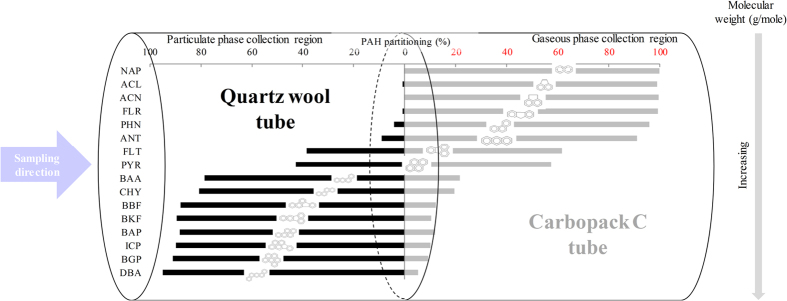
Schematic of the relationship between the QC sampler (combination of quartz wool (QW) and Carbopack C (CC) tubes) and collected PAHs (between particulate and gaseous phases).

Calibration and QA experiments were performed using the CC tube loaded with known quantities of the L-WS containing PAHs. For analysis of real PAH samples in ambient air, collection was performed using serially connected QW and CC tubes (Fig. 3). The resulting tube was called the “QC” tube in order to represent a combination of “QW and CC” in the sampling stage. The front and back fractions of QC were thus used to collect the particulate and gaseous PAHs, respectively (Fig. 4).

### Instrumental system

The analysis of PAH samples in this work was carried out using a GC (model: GC-2010, Shimadzu, Japan) connected to an MS (model: GCMS-QP2010 ultra, Shimadzu, Japan) and a thermal desorber (model: TD-20, Shimadzu, Japan). The schematic and operational conditions of the TD system were set to maximize PAH recovery by virtually eliminating the long transfer line for carrying thermally desorbed PAH from the TD to the GC-MS. The PAHs loaded on the ST were thermally desorbed at 290 °C (7 min) at a reverse flow of 100 mL·min^−1^ of helium (>99.9999%) carrier gas. The desorbed analytes were swept into the cold trap (held at 5 °C) in the stream of carrier gas. The cold trap packed with quartz wool (10 mg) and Tenax TA (50 mg) in a Silcosteel holder (Shimadzu, Japan) was then rapidly desorbed (300 °C for 5 min) in a reverse flow of carrier gas in order to transfer (inject) the target PAHs into the column (DB-5ms - length: 30 m, diameter: 0.25 mm, and thickness: 0.25 μm, Agilent, USA). The transfer/injection of analytes from the cold trap into the GC column was carried out by splitting the flow between the column (2 mL·min^−1^) and the split vent (10 mL·min^−1^). The oven temperature was initially set at 80 °C (for 5 min), ramped at 20 °C·min^−1^ to 300 °C, and held at this temperature for 24 min (a total run time of 40 min).

To detect all 16 target PAHs, the interface and ion source temperatures were set relatively high (e.g., at 280 °C) in order to prevent contamination in the MS system. The PAHs were initially analyzed in total ion chromatographic (TIC) mode over a mass range of 35 to 600 m/z. Extracted ion chromatographic (EIC) mode was subsequently applied to minimize interference and to maximize the sensitivity using significant ions identified from the spectrum of each PAH (Table S5). Detailed information on the instrumental system is included in Table 1.

### Experimental approaches

For the calibration and QA-related experiments, the L-WS containing 16 target PAHs was injected directly into the CC tube and analyzed using the TD-GC-MS system (Figure S3). The CC tube is stronger adsorbent than QW tube. As the analysis of CC tube is expected to show the maximum recovery of PAH, our calibration exp was conducted by CC tube only^27^. The inlet of the CC tube was connected to a gas cylinder containing ultra-pure nitrogen (>99.999%). A Teflon tube was used to connect the ST and the gas cylinder. Then, 1 μL of the L-WS was injected onto the CC tube via a temporary injection port made from the Teflon tube that connected the inlet of the CC tube and the gas cylinder. The nitrogen gas in the gas cylinder was then delivered to the CC tube (flow rate = 3 L·min^−1^ for 3 min). The PAHs loaded on the CC tube were then desorbed using the TD system prior to separation by the GC and final detection by the MS.

The collection of PAH in real ambient air samples was carried out by two-stage sampling with the aid of the QC sampler. This sampler was created as a combination of serially connected QW (front) and CC (back) tubes for collecting particulate and gaseous PAHs, respectively (Fig. 3). The particulate PAHs were first introduced into the QW tube placed at the front, and the gaseous PAHs penetrating the QW tube were then collected by the CC tube (Fig. 4). The outlet of each QC tube was connected to the vacuum pump that interfaced with a mass flow controller (MFC) (Sibata ΣMP-300, Japan). To measure the gas/particle fractionation of PAHs in air, two types of ST (QW and CC) were used simultaneously as PAH sampling media. However, they were desorbed individually for the analysis of PAH partitioned to each individual fraction. The PAH sampling from outdoor air was conducted on the seventh floor (about 21 m) of Jae Sung Engineering Building (HanYang University, Seoul, Korea) for a period of five consecutive days (7 to 11 Oct. 2014). The collection of PAH samples continued for 12 hours starting at midnight (flow rate = 2 L·min^−1^ and total sampling volume = 1.44 m^3^) (Fig. 3). For QC tubes, five code numbers, 1, 2, 3, 4, and 5, were assigned to samples obtained for each of five days. The sample codes were further categorized by assigning a number (order of day) and tube type (QW or QC) such as QW (QW-1, QW-2, QW-3, QW-4, and QW-5) and CC (CC-1, CC-2, CC-3, CC-4, and CC-5). In addition, to test the reliability of QC sampling, triplicate samples of ambient PAHs were simultaneously collected using three QC samplers (Set code: A, B, and C) on 7 Sept. 2014 (sample codes: QW (QW-A, QW-B, and QW-C) and CC (CC-A, CC-B, and CC-C)).

In ST analysis, the breakthrough (BT) volume is one of the key criteria for accurate quantification. In this study, a total volume of 1.44 m^3^ ambient sample was collected at 2 L·min^−1^ for 12 hours. The occurrence of BT on the CC tube was examined by increasing the sampling volume of ambient PAH. To this end, the third calibration point of the L-WS (BAP = 4.98 ng·μL^−1^) was injected onto the CC tube and purged with nitrogen gas up to 2,520 L at a fixed flow rate of 3 L·min^−1^ (six volumes tested between 1 and 2,520 L). The procedures for BT test using the L-WS were adopted from those used for the analysis of the L-WS but at varying purge volumes (by controlling the purge times). After this purge stage, the CC tubes were analyzed using a TD-GC-MS system.

## Additional Information

**How to cite this article**: Kim, Y.-H. and Kim, K.-H. A simple methodological validation of the gas/particle fractionation of polycyclic aromatic hydrocarbons in ambient air. *Sci. Rep.* **5**, 11679; doi: 10.1038/srep11679 (2015).

## Supplementary Material

Supplementary Information

## Figures and Tables

**Table 1:** Operational conditions for the analysis of 16 target PAHs using the TD-GC-MS system in this work.

**[A] Thermal desorber (model: TD-20, Shimadzu, Japan)**				
*a*. *Sampling tube*				
1. Trap tube:	Quartz (length: 90 mm, OD: 6.4 mm, and ID: 4.2 mm)			
2. Adsorbent:	(1) Quartz wool 25 mg (QW tube);			
	(2) Quartz wool 10 mg + Carbopack C (60/80) 50 mg + Quartz wool 10 mg (CC tube)			
3. Desorption flow:	100 mL·min^−1^ (to cold-trap)			
4. Desorption time:	7 min			
5. Desorption temperature:	290 °C			
*b. Cold-trap*				
1. Trap tube:	Silcosteel (length: 100 mm, OD: 3.2 mm, and ID: 2 mm)			
2. Adsorbent:	Quartz wool 10 mg + Tenax TA 50 mg			
3. Adsorption temperature:	5 °C (from sampling tube)			
4. Desorption temperature:	300 °C (to GC)			
5. Desorption flow:	16 mL·min^−1^			
*c. Carrier gas setting*				
1. Carrier gas:	Helium (>99.999%)			
2. Constant gas flow:	2 mL·min^−1^			
3. Split flow:	10 mL·min^−1^			
4. Purge gas flow:	2 mL·min^−1^			
*d. Line and interface temperatures: 300 °C*				
**[B] Gas chromatography (model: GC-2010, Shimadzu, Japan)**				
a. Column:	DB-5ms (Agilent J&W, USA)			
	(length (30 m), diameter (0.25 mm), and film thickness (0.25 μm))			
b. Oven settings:	80 °C (5 min) → 20 °C/min → 300 °C (24 min)			
	(Total program time = 40 min)			
**[C] Mass spectrometry (model: GCMS-QP2010 ultra, Shimadzu, Japan)**				
a. Ionization mode:	EI (70 eV)	d. TIC scan range:	35 ~ 600 m/z	
b. Ion source temperature:	280 °C	e. Scan speed:	1250	
c. Interface temperature:	280 °C			

**Table 2:** Comparison of detection limits and pretreatment procedures for PAH analysis in different studies.

Order	Method	Detection limit (pg)				Pretreatment procedures				Ref.
	Code^a^	Mean ± SD		Min–Max		Used filter	Extracted	Injection	Expected	
		MDL	LOD	MDL	LOD	area (%)	volume (μL)	volume (μL)	recovery (%)	
1	T-AbBd-4-α	13.1 ± 7.04	0.76 ± 0.20	5.19–27.0	0.57–1.23	100	—	—	100	**This study (2014)**
2	T-Aa-4-α		122 ± 69.0			25	—	—	25	Bates *et al.*^18^
3	T-AcBe-4-α		1.86 ± 0.79		0.86–3.74	100	—	—	100	Wauters *et al.*^19^
4	T-AcBe-4-α		67.9 ± 83.5		1.44–259	100	—	—	100	Lazarov *et al.*^20^
5	C-Ab-1-α	208 ± 138		66.2–497		50	100	2	1	Bari *et al.*^15^
6	C-AbBd-1-α			9.21–25.3		100	1,000	2	0.2	Ma *et al.*^13^
7	C-AbBd-1-α				6.9–157	100	500	1	0.2	Sheu *et al.*^28^
8	C-AaBd-1-α			200–1,000		100	1,000	1	0.1	Anthwal *et al.*^14^
9	C-AaBd-1-γ				0.58–7.99	100	1,000	20	2	Albinet *et al.*^17^
10	C-Aa-1-γ		2.09 ± 1.11		0.70–4.30	0.28	500	20	0.011	Okuda *et al.*^16^
11	C-AbBd-1-β		330 ± 116		220–440	100	500	10	2	Yamasaki *et al.*^29^
12	C-Aa-3-α		566 ± 336		20–1,160	100	—	—	—	Menezes and de Lourdes Cardeal^30^
13	C-Ac-2-γ		7.69 ± 4.54		2.51–14.8	100	1,000	20	2	Schnelle-Kreis *et al.*^31^

^a^Method code: Method type-Phase/Sampler-Pretreatment-Separation/Detection (refer to the Fig. 1).

**Table 3:** Overview of sampling conditions for PAHs with several types of analytical methods.

Order	Method	No. of		Sampling conditions						Ref.
	Code^a^	target		Pump^b^	Site	Period	Flow rate	Time	Volume	
		PAHs	Rings			(starting time)	(m^3^·min^−1^)	(hr)	(m^3^)	
1	T-AbBd-4-α	16	2–6	Sibata MP300	Seoul, Korea (7th building)	7 ~ 11 Oct. 2014 (00:00)	0.002	12	1.44	This study (2014)
2	T-Aa-4-α	9	4–6	LVAS	Bari, Italy (street with traffic)	NI^c^	0.01667	24	24	Bates, *et al.*^18^
3	T-AcBe-4-α	16	2–6	Gilair 3 personal air sampling pump	Ghent, Belgium (Lab campus)	Apr. 2005 ~ Mar. 2006	0.0001	24	0.144	Wauters *et al.*^19^
4	T-AcBe-4-α	16	2–6	GSA SG350	Antwerp, Belgium	Mar. ~ May 2012	0.000333	24	480	Lazarov *et al.*^20^
5	C-Ab-1-α	21	2–6	LVAS	Dettenhausen, Germany	1 Nov. 2005 ~ 31 Mar 2006	0.038	48 and 72	110 and 166	Bari, *et al.*^15^
6	C-AbBd-1-α	16	2–6	HVAS	Harbin, China (Northeastern)	5 Aug. 2008 ~ 29 July 2009	0.8	24	1,152	Ma, *et al.*^13^
7	C-AbBd-1-α	21	2–6	HVAS	Center of Tainan City, Taiwan	21 Jan. ~ 25 May 1994	0.69	24	1,000	Sheu, *et al.*^28^
8	C-AaBd-1-α	17	2–6	HVAS	Seoul, Korea	Feb. ~ July 2009	0.8	24	1,152	Anthwal, *et al.*^14^
9	C-AaBd-1-γ	15	3–7	HVAS	Marseilles area, South of France	22 ~ 29 July 2004 (08:00)	0.5	12	360	Albinet, *et al.*^17^
10	C-Aa-1-γ	16	3–7	HVAS	Beijing, China	Sept. 2003 ~ Apr. 2005 (11:00)	0.8	24	1,152	Okuda, *et al.*^16^
11	C-AbBd-1-β	17	3–6	HVAS	Osaka, Japan	7, Nov. 1977 ~ 9, Nov. 1978	0.75	24	1,080	Yamasaki, *et al.*^29^
12	C-Aa-3-α	16	2–6	HVAS	Divinopolis and Minas Gerais, Brazil	Nov. 2009	1.03	24	1,488	Menezes and de Lourdes Cardeal^30^
13	C-Ac-2-γ	7	3–6	LPI	Munich, Germany	1996 ~ 1998	0.02971	23.5	42	Schnelle-Kreis, *et al.*^31^
14	C-AbBd-1-α	11	3–6	HVAS	Chicago and Michigan, USA	July 1994 (08:00)	0.5–0.8	12	360–576	Simcik, *et al.*^21^
15	C-AbBd-1-α	16	2–6	HVAS	Beijing, China	6 Sept. 2008 ~ 29 July 2009	0.8	24	1,152	Ma, *et al.*^22^

^a^Method code: Method type-Phase/Sampler-Pretreatment-Separation/Detection (refer to the Fig. 1).

^b^LVAS = low-volume air sampler; HVAS = high-volume air sampler; low = pressure cascade impactor.

^c^No information.

**Table 4:**
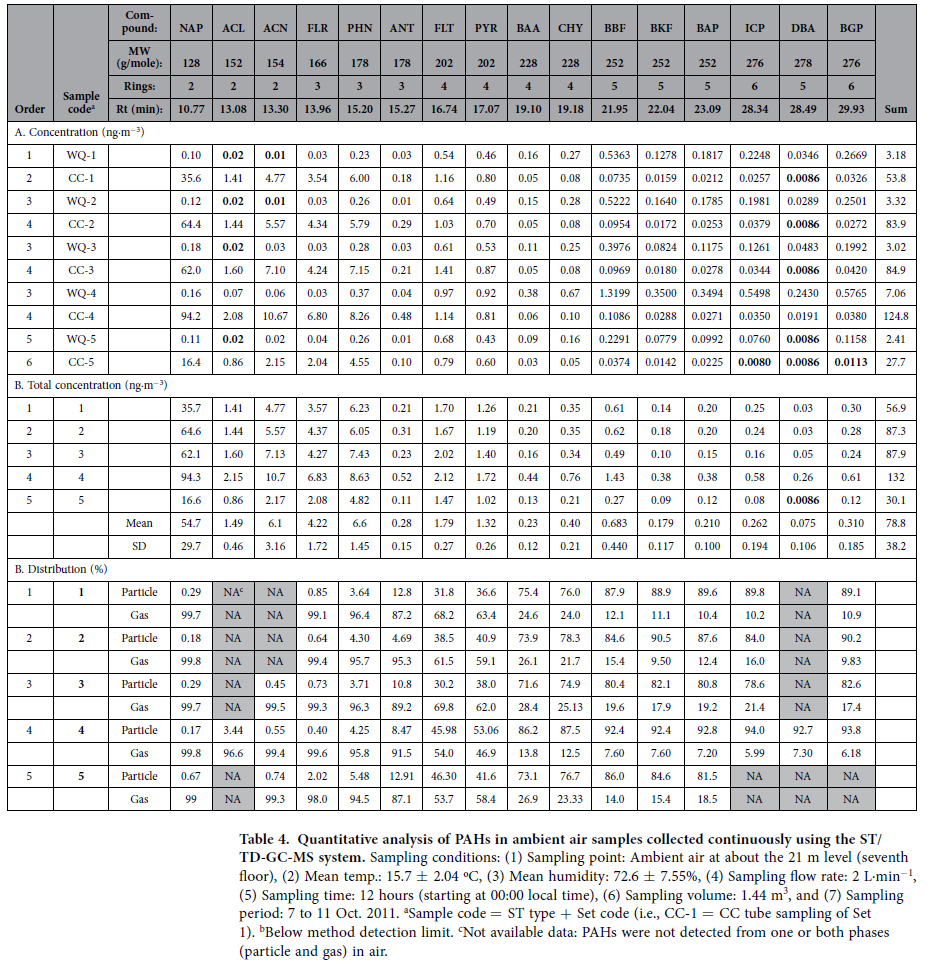
Quantitative analysis of PAHs in ambient air samples collected continuously using the ST/TD-GC-MS system.

**Table 5:**
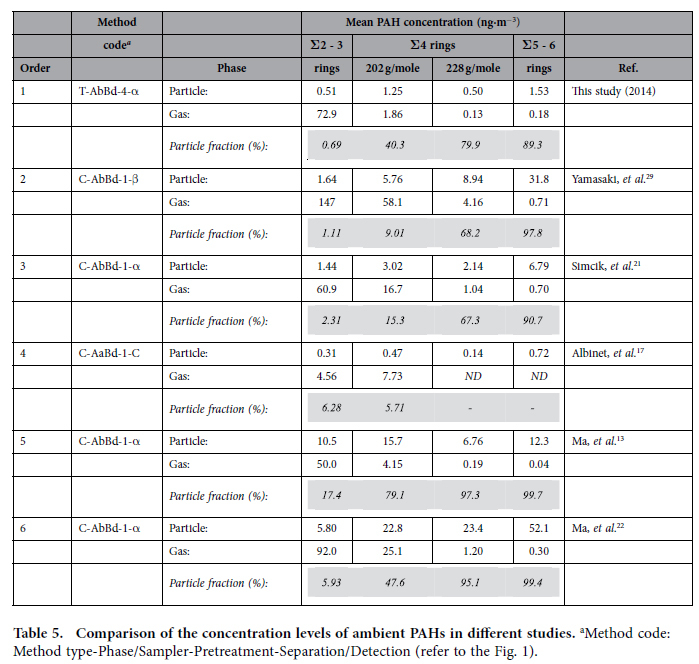
Comparison of the concentration levels of ambient PAHs in different studies.
