# Supplementary material for: A simple methodological validation of the gas/particle fractionation of polycyclic aromatic hydrocarbons in ambient air
Source: Sci Rep. 2015 Jul 1;5:11679. doi: 10.1038/srep11679 (PMC4486962; doi:10.1038/srep11679)
Supplement: Supplementary Information [file srep11679-s1.pdf]

13 May 2015 (Scientific Reports_SI_R1_V3)

Supplementary Information

A simple methodological validation of the gas/particle fractionation of polycyclic aromatic hydrocarbons in ambient air

Yong-Hyun Kim, Ki-Hyun Kim*

Department of Civil and Environmental Engineering, Hanyang University,

222 Wangsimni-Ro, Seoul 133-791, Korea

* Corresponding author: kkim61@hanyang.ac.kr or kkim61@nate.com, Phone: 82-2-2220-2325. Fax -1945

Previously at: Dept. of Energy & Environment, Sejong University, Seoul, Korea

Supplementary figure legends

Figure S1 | Plot of the mean concentrations of 16 PAHs in ambient air measured continuously at the Jae Sung Engineering Building (seventh floor), HanYang University, Seoul, Korea (7 to 11 Sept. 2014).

Figure S2 | Relative fractionation (%) patterns of 16 PAHs in ambient air samples between particulate and gaseous phases: Occurrences of PAH in three different phase boundaries: (a) gaseous phase dominance, (b) dynamic fractionation between gas and particulate phases, and (c) particulate phase dominance.

Figure S3 | Experimental procedure for loading the liquid-phase PAH standards onto the CC tube through direct injection.

Table S1 | Results of calibration experiments using liquid phase standards of 16 PAHs: (1) RF, (2) R^2, and (3) RSE.

Order	Compound	MW (number of rings)	[A] Response factor (ng^{-1})	[B] Coefficient of determination (R^2)	[C] Relative standard error[a] (RSE, %)
1	NAP	128 (2)	**31,060**	0.9967	0.61
2	ACL	152 (2)	**23,361**	0.9953	0.54
3	ACN	154 (2)	**23,707**	0.9995	0.69
4	FLR	166 (3)	**21,084**	0.9953	0.63
5	PHN	178 (3)	**38,336**	0.9994	0.28
6	ANT	178 (3)	**38,014**	0.9998	0.11
7	FLT	202 (4)	**37,326**	0.9995	0.49
8	PYR	202 (4)	**40,957**	0.9996	0.47
9	BAA	228 (4)	**40,458**	0.9990	0.94
10	CHY	228 (4)	**42,691**	0.9992	0.36
11	BBF	252 (5)	**39,524**	0.9991	0.60
12	BKF	252 (5)	**40,992**	0.9990	0.75
13	BAP	252 (5)	**39,957**	0.9991	0.74
14	ICP	279 (6)	**36,249**	0.9993	0.80
15	DBA	278 (5)	**45,466**	0.9988	0.79
16	BGP	276 (6)	**37,053**	0.9999	0.30

[a]Triplicate analyses of the liquid standards prepared for the third calibration point (injection volume = 1 µL)

Table S2 | The results of detection limit analysis for 16 target PAHs based on sorbent tube analysis in this research.

Order	Compound	MW (rings)	MDL[a]		LOD[b]	
			(pg)	(pg/m^3)[c]	(pg)	(pg/m^3)[c]
1	NAP	128 (2)	26.4	18.3	0.84	0.58
2	ACL	152 (2)	27.0	18.7	1.11	0.77
3	ACN	154 (2)	17.8	12.4	1.10	0.76
4	FLR	166 (3)	23.0	16.0	1.23	0.86
5	PHN	178 (3)	9.41	6.54	0.68	0.47
6	ANT	178 (3)	8.35	5.80	0.68	0.47
7	FLT	202 (4)	8.20	5.70	0.70	0.48
8	PYR	202 (4)	11.6	8.02	0.63	0.44
9	BAA	228 (4)	6.51	4.52	0.64	0.45
10	CHY	228 (4)	7.91	5.49	0.61	0.42
11	BBF	252 (5)	11.0	7.64	0.66	0.46
12	BKF	252 (5)	5.19	3.60	0.63	0.44
13	BAP	252 (5)	6.62	4.60	0.65	0.45
14	ICP	279 (6)	11.5	7.96	0.72	0.50
15	DBA	278 (5)	12.4	8.59	0.57	0.40
16	BGP	276 (6)	16.2	11.3	0.70	0.49
	Mean		13.1	9.07	0.76	0.53
	SD		7.04	4.89	0.20	0.14

[a]The product of the standard deviation of seven replicates multiplied by the Student's t-value at the 99.9% confidence level (6 df, t=3.14); 1 μL injection of 16 PAH solution prepared by 25-fold dilution of the first calibration point of L-WS with methanol

[b]Calculated using three times the standard deviation of seven background noises

[c]Assuming a total sample volume of 1.44 m^3

Table S3 | Results of breakthrough test for PAH analysis according to sorbent tube method: Comparison of mass recovery (%) in the CC tube with varying purge gas conditions.

Order	MW (rings)	Compound		Relative recovery[a] (%)					
			Sweep gas volume (L):	1	9	45	90	540	2,520
1	128 (2)	NAP		63.8	99.5	98.5	97.8	102	100
2	152 (2)	ACL		75.5	99.9	98.7	97.1	98.4	99.4
3	154 (2)	ACN		75.4	97.7	94.1	95.5	100	98.8
4	166 (3)	FLR		75.3	99.7	99.4	99.7	99.8	98.8
5	178 (3)	PHN		69.6	99.7	97.8	97.6	96.3	99.1
6	178 (3)	ANT		78.2	99.7	96.8	99.0	96.9	95.8
7	202 (4)	FLT		75.4	99.7	99.8	94.6	99.1	98.4
8	202 (4)	PYR		73.1	99.7	97.1	93.9	101	99.4
9	228 (4)	BAA		76.6	99.6	96.5	101	104	102
10	228 (4)	CHY		77.0	99.6	97.1	99.2	102	98.2
11	252 (5)	BBF		70.2	99.7	95.2	96.9	98.5	99.3
12	252 (5)	BKF		71.5	99.6	96.1	102	100	98.4
13	252 (5)	BAP		70.9	99.6	97.4	99.8	101	101
14	279 (6)	ICP		70.7	99.6	97.6	97.8	98.5	98.8
15	278 (5)	DBA		68.2	99.6	96.7	100	99.7	99.5
16	276 (6)	BGP		69.4	99.7	98.5	101	97.6	99.0

[a] Relative recovery (%) = Measured mass (ng) / Injected mass (ng) * 100

Measured mass (ng) = Peak area / RF value (ng^{-1})

The L-WS (third calibration point) was injected onto the ST, while the purge gas flowed from the nitrogen cylinder (> 99.999%) equipped with Carbopack X filter (50 mg) to the ST (flow rate = 3 L min^{-1})

Table S4 | Analysis of ambient PAHs in air using the ST/TD-GC-MS system based on triplicate analyses in order to assess the reproducibility of the ST sampling.

Order	Sample code[a]	Compound:	**NAP**	**ACL**	**ACN**	**FLR**	**PHN**	**ANT**	**FLT**	**PYR**	**BAA**	**CHY**	**BBF**	**BKF**	**BAP**	**ICP**	**DBA**	**BGP**	**Sum**
		MW (g/mol):	128	152	154	166	178	178	202	202	228	228	252	252	252	276	278	276	
		Rings:	2	2	2	3	3	3	4	4	4	4	5	5	5	6	5	6	
		Rt (min)	10.77	13.08	13.30	13.96	15.20	15.27	16.74	17.07	19.10	19.18	21.95	22.04	23.09	28.34	28.49	29.93	
**A. Concentration (ng·m^{-3})**																			
1	WQ-A		**0.02**[b]	**0.02**	**0.01**	0.03	0.32	0.04	0.89	0.53	0.08	0.12	0.2148	0.0551	0.0720	0.0731	0.0309	0.1261	2.58
2	CC-A		69.0	2.63	12.2	3.27	13.0	1.04	4.15	1.70	0.01	0.01	0.0100	0.0040	**0.0046**	**0.0080**	**0.0086**	**0.0113**	107
3	WQ-B		**0.02**	**0.02**	**0.01**	0.02	0.15	0.03	0.48	0.28	0.06	0.08	0.1263	0.0310	0.0601	0.0469	0.0171	0.0667	1.44
4	CC-B		50.6	2.79	10.97	3.35	11.9	1.01	4.52	1.92	0.01	0.01	**0.0076**	**0.0036**	**0.0046**	**0.0080**	**0.0086**	**0.0113**	87.0
5	WQ-C		**0.02**	**0.02**	**0.01**	**0.02**	0.12	0.01	0.32	0.20	0.06	0.08	0.1262	0.0349	0.0547	0.0596	0.0247	0.0703	1.16
6	CC-C		44.4	2.56	9.98	3.30	11.5	0.71	2.94	1.07	0.01	0.01	**0.0076**	**0.0036**	**0.0046**	**0.0080**	**0.0086**	**0.0113**	76.5
**B. Total concentration (ng·m^{-3})**																			
1	A		69.0	2.63	12.2	3.31	13.3	1.08	5.04	2.23	0.09	0.13	0.225	0.059	0.072	0.073	0.031	0.126	110
2	B		50.6	2.79	11.0	3.37	12.0	1.04	4.99	2.19	0.07	0.09	0.126	0.031	0.060	0.047	0.017	0.067	88.4
3	C		44.4	2.56	10.0	3.30	11.6	0.72	3.25	1.28	0.06	0.09	0.126	0.035	0.055	0.060	0.025	0.070	77.6
		Mean	54.6	2.66	11.0	3.33	12.3	0.95	4.43	1.90	0.07	0.10	0.159	0.042	0.062	0.060	0.024	0.088	91.9
		SD	12.8	0.12	1.11	0.04	0.87	0.20	1.02	0.54	0.02	0.03	0.057	0.015	0.009	0.013	0.007	0.033	16.2
**B. Distribution (%)**																			
1	**A**	Particle	NA[c]	NA	NA	1.05	2.38	3.44	17.7	23.8	89.6	92.2	95.5	93.1	NA	NA	NA	NA	
		Gas	NA	NA	NA	99.0	97.6	96.6	82.3	76.2	10.4	7.84	4.45	6.85	NA	NA	NA	NA	
2	**B**	Particle	NA	NA	NA	0.71	1.28	2.58	9.57	12.60	89.7	92.6	NA	NA	NA	NA	NA	NA	
		Gas	NA	NA	NA	99.3	98.7	97.4	90.4	87.4	10.3	7.42	NA	NA	NA	NA	NA	NA	
3	**C**	Particle	NA	NA	NA	NA	1.04	1.87	9.72	16.1	88.8	92.8	NA	NA	NA	NA	NA	NA	
		Gas	NA	NA	NA	NA	99.0	98.1	90.3	83.9	11.2	7.16	NA	NA	NA	NA	NA	NA	

[a]Sample code = ST type + Set code (i.e., QW-A = QW tube sampling of Set A)

Sampling conditions: (1) Sampling point: Ambient air at about the 21 m level (seventh floor), (2) Mean temp.: 21.0 ± 1.23 ℃, (3) Mean humidity: 66.2 ± 4.49%, (4) Sampling flow rate: 2 L·min^{-1}, (5) Sampling time: 12 hours (starting at 00:00 local time), (6) Sampling data: 14 Sept. 2014, and (7) Sampling volume: 1.44 m^{3}

[b]**Below method detection limit**

[c]Not available data: PAHs were not detected from one or both phases (particle and gas) in air

Table S5 | List of 16 target polycyclic aromatic hydrocarbons (PAHs) selected for the TD-based analysis in this research.

Order	Compounds	Short name	Number of aromatic rings	MW ($g \cdot mol^{-1}$)	Density ($g \cdot cm^{-3}$)	Melting point (℃)	Boiling point (℃)	Formula	CAS number	Mass spectra[a] (m/z)
1	Naphthalene	NAP	2	128	1.14	80.26	218	$C_{10}H_{18}$	91-20-3	128
2	Acenaphthylene	ACL	2	152	0.90	91.8	280	$C_{12}H_8$	208-96-8	152
3	Acenaphthene	ACN	2	154	1.22	93.4	279	$C_{12}H_{10}$	83-32-9	154
4	Fluorene	FLR	3	166	1.20	116	295	$C_{13}H_{10}$	86-73-7	166
5	Phenanthrene	PHN	3	178	1.18	101	332	$C_{14}H_{10}$	85-01-8	178
6	Anthracene	ANT	3	178	1.25	218	340	$C_{14}H_{10}$	120-12-7	178
7	Fluoranthene	FLT	4	202	1.25	110.8	375	$C_{16}H_{10}$	206-44-0	202
8	Pyrene	PYR	4	202	1.27	145	404	$C_{16}H_{10}$	129-00-0	202
9	Benz[a]anthracene	BAA	4	228	1.19	158	340	$C_{18}H_{12}$	56-55-3	228
10	Chrysene	CHY	4	228	1.27	254	448	$C_{18}H_{12}$	218-01-9	228
11	Benzo[b]fluoranthene	BBF	5	252	1.29	168	481	$C_{20}H_{12}$	205-99-2	252
12	Benzo[k]fluoranthene	BKF	5	252	1.29	217	480	$C_{20}H_{12}$	207-08-9	252
13	Benzo[a]pyrene	BAP	5	252	1.24	179	495	$C_{20}H_{12}$	50-32-8	252
14	Indeno[1,2,3-c,d]pyrene	ICP	6	276	1.38	163	536	$C_{22}H_{12}$	193-39-5	276
15	Dibenz[a,h]anthracene	DBA	5	278	1.23	262	524	$C_{22}H_{14}$	53-70-3	278
16	Benzo[g,h,i]perylene	BGP	6	276	1.38	278	500	$C_{22}H_{12}$	191-24-2	276

[a]Mass spectra selected for the EIC-based analysis

Table S6 | Basic information for the preparation of 16 PAH mix standard for the TD-based calibration analysis.

Order	Compounds	16 PAH mixture[a] Concentration (μg·mL^{-1})	16 PAH mixture[a] Purity (%)	L-WS[b] (ng·μL^{-1}) Six-point calibration values 1st	2nd	3rd	4th	5th	6th
**[A] Concentration (or purity)**									
1	NAP	1000	99.9	12.5	25.0	50.0	125	250	500
2	ACL	2000	99.2	24.8	49.6	99.2	248	496	992
3	ACN	1001	99.9	12.5	25.0	50.0	125	250	500
4	FLR	200.8	99.1	2.49	4.97	9.95	24.9	49.7	99.5
5	PHN	100.4	99.0	1.24	2.48	4.97	12.4	24.8	49.7
6	ANT	99.6	99.1	1.23	2.47	4.94	12.3	24.7	49.4
7	FLT	199.2	98.3	2.45	4.90	9.79	24.5	49.0	97.9
8	PYR	100.4	98.8	1.24	2.48	4.96	12.4	24.8	49.6
9	BAA	100.0	99.9	1.25	2.50	5.00	12.5	25.0	50.0
10	CHY	100.4	99.9	1.25	2.51	5.01	12.5	25.1	50.1
11	BBF	200.0	98.7	2.47	4.94	9.87	24.7	49.4	98.7
12	BKF	100.4	99.9	1.25	2.51	5.01	12.5	25.1	50.1
13	BAP	100.0	99.6	1.25	2.49	4.98	12.5	24.9	49.8
14	ICP	99.6	99.6	1.24	2.48	4.96	12.4	24.8	49.6
15	DBA	199.6	99.8	2.49	4.98	10.0	24.9	49.8	100
16	BGP	199.2	99.2	2.47	4.94	9.88	24.7	49.4	98.8
**[B] Mixing recipe: in volume (μL)[c]**									
			16 PAH mixture	25	50	100	250	500	1,000
			MeOH	1,975	1,950	1,900	1,750	1,500	1,000
			Total	2,000	2,000	2,000	2,000	2,000	2,000

[a]EPA 610 Polynuclear Aromatic Hydrocarbons Mixture (Supelco, St. Louis, MO, USA)

[b]The liquid working standard (L-WS): Constant injection volume of 1 μL for the ST-based calibration analysis

[c]Mixing conditions and formula for L-WS are given

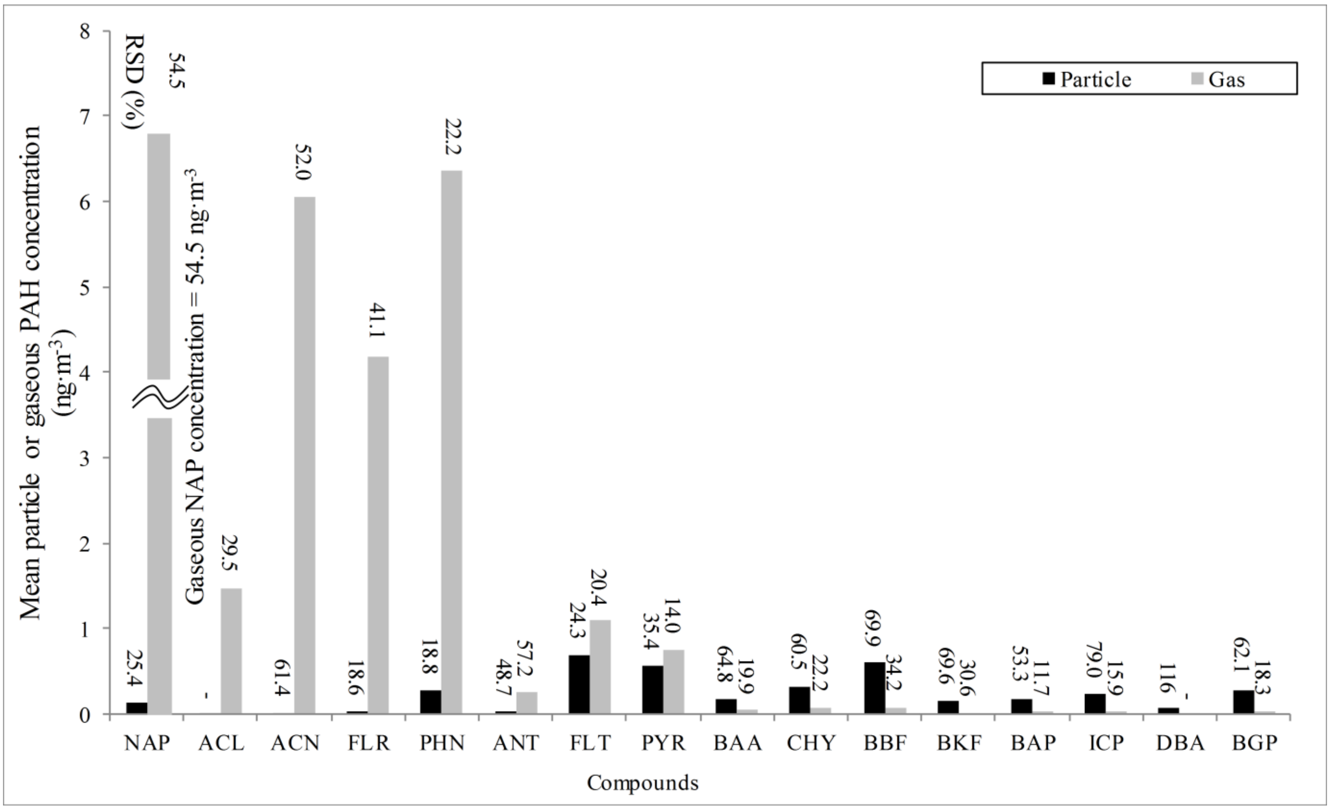

Figure S1 | Plot of the mean concentrations of 16 PAHs in ambient air measured continuously at the Jae Sung Engineering Building (seventh floor), HanYang University, Seoul, Korea (7 to 11 Sept. 2014)

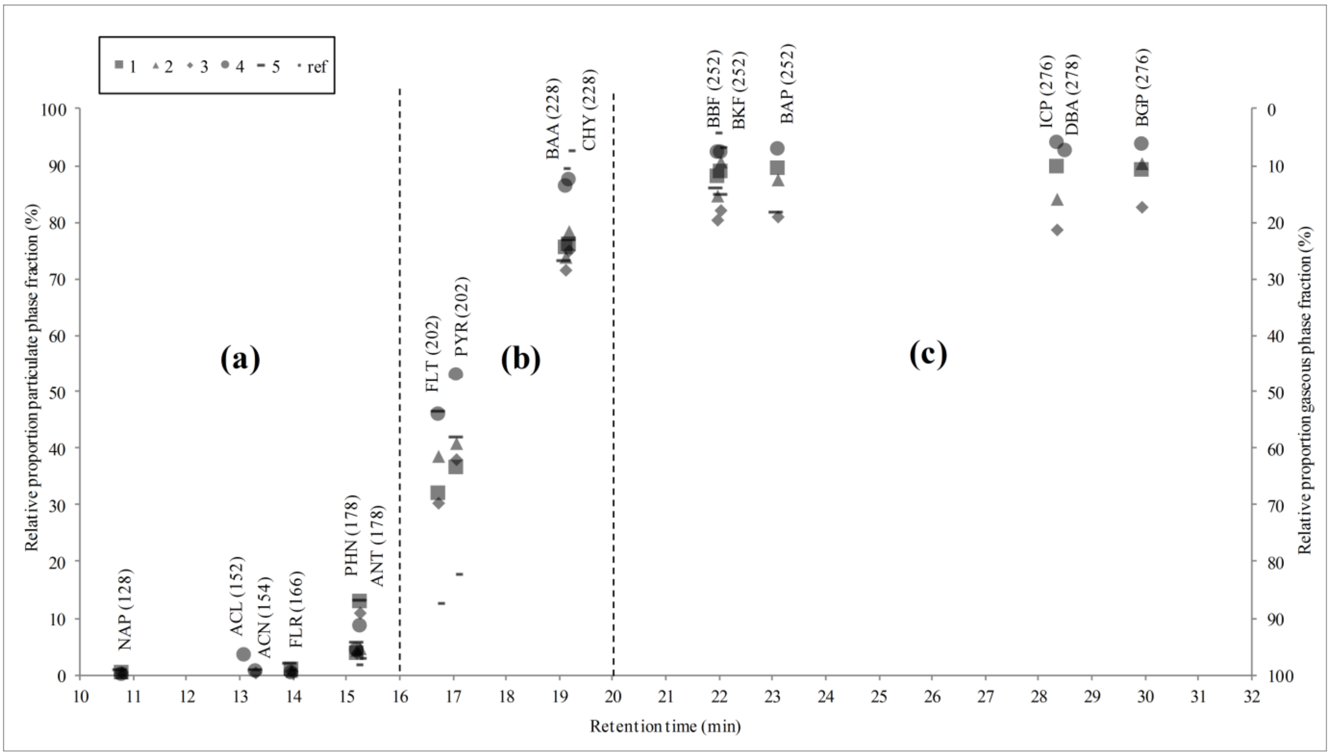

Figure S2 | Relative fractionation (%) patterns of 16 PAHs in ambient air samples between particulate and gaseous phases: Occurrences of PAH in three different phase boundaries: (a) gaseous phase dominance, (b) dynamic fractionation between gas and particulate phases, and (c) particulate phase dominance.

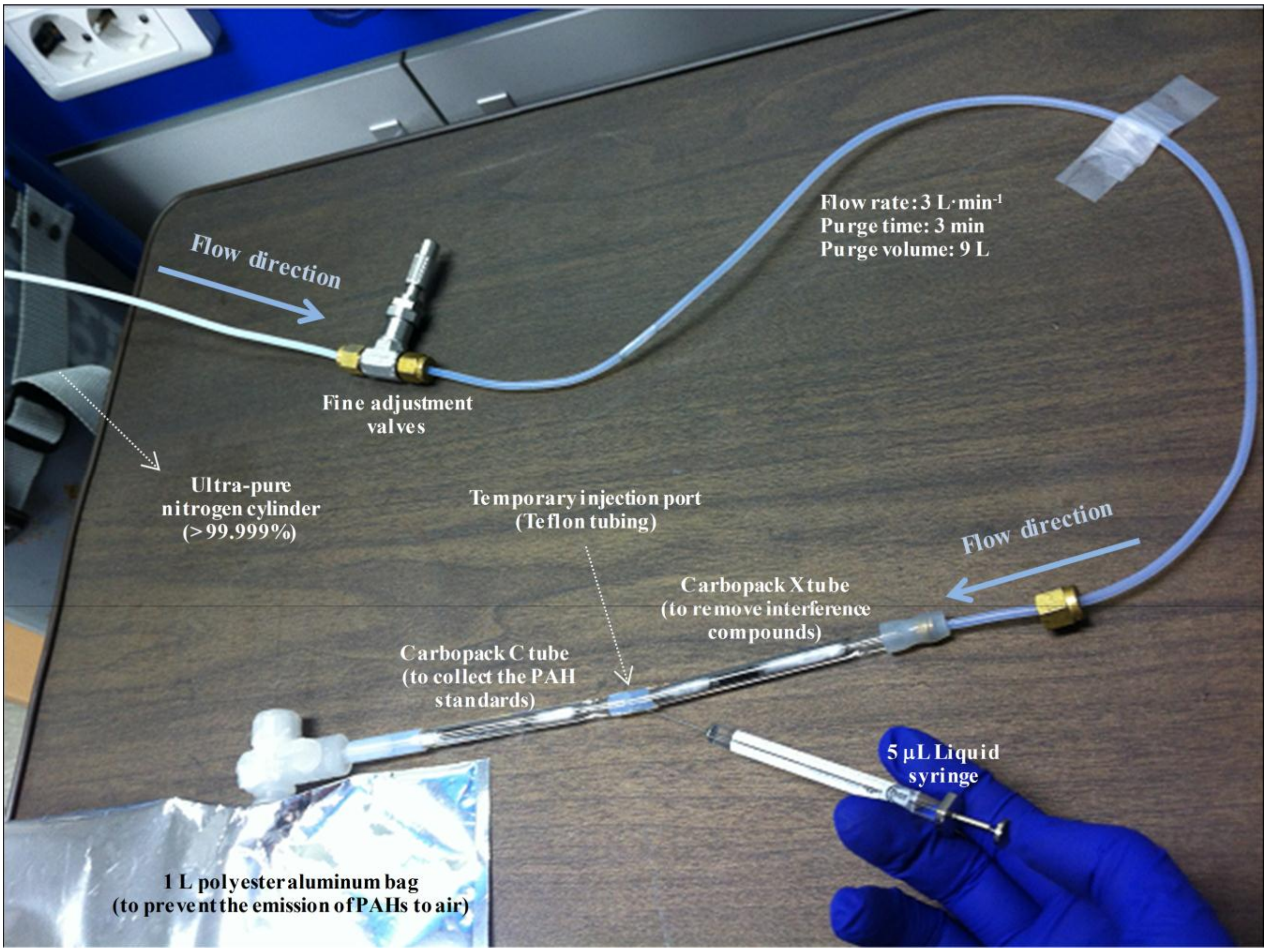

Figure S3 | Experimental procedure for loading the liquid-phase PAH standards onto the CC tube through direct injection
